# Bacterial translocation primes proinflammatory responses and is connected to early death in an experimental model of lethal injury

**DOI:** 10.1186/cc11954

**Published:** 2013-03-19

**Authors:** N Baxevanos, T Tsaganos, A Pistiki, D Droggiti, A Spyridaki, E Giamarellos-Bourboulis

**Affiliations:** 1 University of Athens, Medical School, Athens, Greece

## Introduction

Some cases of multiple trauma are rapidly deteriorating; the mechanism was investigated.

## Methods

Forty-one rabbits were assigned into two groups; sham-operated and subject to crush of the right femur. Survival was recorded; peripheral blood was sampled for LPS measurement by the kinetic QCL-1000 LAL assay; quantitative tissue growth was assessed after death. Some rabbits were sacrificed at 48 hours; blood was sampled from the portal vein for LPS measurement; splenocytes were isolated and incubated for 24 hours in the presence of 10 ng/gl LPS of *Escherichia coli *O55:B5 and of 5 μg/ml phytohemagglutin (PHA); TNFα was measured in supernatants by a bioassay on L929 fibroblasts.

## Results

**Figure 1:**
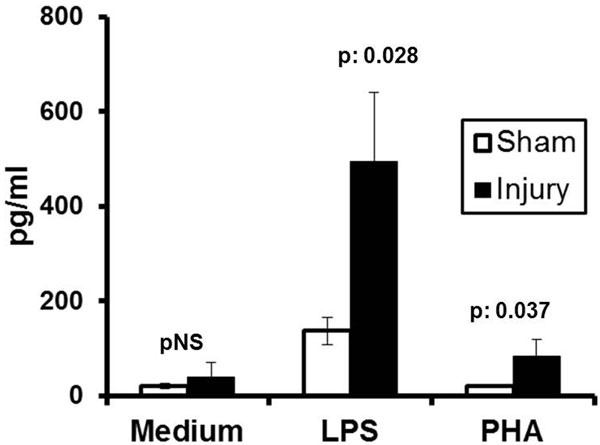
**Stimulation of TNFα from isolated splenocytes**.

Fifty percent of rabbits died early; that is, within the first 48 hours. Mean ± SE log_10 _bacteria in the liver and lung of animals that died early was 2.27 ± 0.62 and 3.16 ± 0.783cfu/g; respective values of rabbits that started dying late (that is, after 72 hours) were below the limit of detection. Mean circulating LPS at 24 hours was 2.09 EU/ ml and 1.99 EU/ml respectively (*P *= NS). Mean LPS of the portal vein of the sham and of the injury groups were 1.25 and 5.62 EU/ml (*P *= 0.047). Concentrations of TNFα in splenocyte supernatants are shown in Figure 1.

## Conclusion

Early death after injury is not related to peripheral endotoxemia and sepsis; bacterial translocation priming for enhanced proinflammatory responses is a likely explanation.

